# Exploring the impact of COVID-19 on reported maternal and neonatal complications and access to maternal health care in five government health facilities in Blantyre, Malawi

**DOI:** 10.1371/journal.pone.0285847

**Published:** 2023-05-23

**Authors:** Alden Blair, Winta Haile, Anna Muller, Luseshelo Simwinga, Richard Malirakwenda, Kimberly Baltzell

**Affiliations:** 1 San Francisco (UCSF) Institute for Global Health Sciences, University of California, San Francisco, CA, United States of America; 2 Global AIDS Interfaith Alliance, Limbe, Malawi; 3 San Francisco (UCSF) School of Nursing, University of California, San Francisco, CA, United States of America; University of the Witwatersrand, SOUTH AFRICA

## Abstract

**Background:**

There is a dearth of studies assessing the effects of SARS-CoV-2 on the healthcare system and access to care, especially in lower- and middle-income countries such as Malawi. We aimed to assess the impacts of COVID-19 on reported maternal and neonatal complications as well as potential changes in maternal care access to care among five primary care health facilities in Blantyre, Malawi.

**Methods:**

This retrospective cohort study assessed maternal and neonatal register data from five participating health centers in Blantyre, Malawi using the Malawi District Health Information Software 2 (DHIS2) to compare outcomes from 15 months before COVID-19 emerged, defined as the pre-Covid period (January 2019 –March 2020) with nine months after COVID-19 (April 2020 –December 2020).

**Results:**

There was a significant decrease in reported use of vacuum extraction, which went from <0.01%in the pre-COVID period to 0% in the COVID period (p = 0.01). The proportion of births reporting fetal distress almost tripled from 0.46% to 1.36% (p = 0.001) during the COVID-19 period. Additionally, reported anticonvulsant use significantly increased from 0.01% to 1.2% (p<0.01), and antibiotic use significantly increased from 0.45% to 1.6% (p = 0.01). Asphyxia was the only significant neonatal complication variable reported, increasing from 2.80% to 3.45% (p = 0.01).

**Conclusion:**

Our findings suggest that significant outcomes were mainly due to the indirect effects of COVID-19 rather than the virus itself. Based on our findings and the contextual qualitative interviews with two Malawian expert midwives, we concluded that mothers may have been affected more due to understaffing and shortage of skilled personnel in the study health facilities. Therefore, the development of highly skilled health workers may contribute to better outcomes, along with adequate staffing and a streamlined referral process.

## Introduction

The severe acute respiratory syndrome coronavirus-2 (SARS-CoV-2) emerged in November 2019 and quickly became a global pandemic. Following its emergence, governments around the world responded to contain its spread by implementing business shutdowns, movement restrictions, and social distancing [1]. The rise in COVID-19 patients required precautionary measures to be implemented, such as nurse shifts in teams to limit virus exposure between nurses, which as a result led to understaffed labor wards and diminished access to and/or attendance of healthcare services [2,3].

Given the need for routine prenatal visits with care providers as a way of identifying and reducing complications, disruptions caused by COVID-19 pandemic may have disproportionally affected pregnant women [4]. Indeed, recent work has highlight links between the disease and non-respiratory complications in birthing women [5,6]. This in turn may have had a significant effect on those birthing parents in low- and middle-income countries (LMICs), who are already at higher risk of maternal and neonatal complications. This was apparent in countries such as Nepal, where the number of births at hospitals decreased by almost 90% over a four-month lockdown as more women opted to deliver at home. As a result, maternal deaths were five times higher, neonatal deaths were three times higher, and stillbirths were 1.5 times higher between March 2020 and June 2021 compared to same period the year before [7,8]. In other LMICs including India, Indonesia, Nigeria, and Pakistan, maternal mortality, newborn death, and stillbirths showed a combined 30% increase since the start of the pandemic [9].

Despite expert predictions, reported COVID-19 cases and deaths were low in sub-Saharan Africa (SSA), compared to other regions of the world [10,11]. The relatively low number of cases and deaths have been attributed to quick action and effective implementation of preventive polices by SSA governments, public support, a young population, and hot climate [12]. There could be several other reasons as to why reported COVID-19 cases are low in SSA, including low detection level due to limited testing laboratory capacity, inadequate testing rates, and late disease introduction compared to the rest of the world [12,13].

Nonetheless, government restrictions to curb the spread of the pandemic affected people’s everyday lives including their incomes and mobility. In Kenya, Uganda, and Tanzania, it was reported that this resulted in limitations of pregnant women’s access to care as well as midwives transport options while trying to conduct facility-based deliveries or home-based deliveries [14]. Additionally, fear of contracting COVID-19 has also kept both pregnant women from hospitals and providers from practicing [14]. Prior epidemics such as the 2013 Ebola outbreak give suggested evidence as to the longer term consequences of these responses. During that pandemic, failures in access to healthcare services resulted in more deaths caused by malaria, measles, HIV/AIDS, and tuberculosis than deaths caused by Ebola [15]. Maternal delivery care also decreased by over 80% in places affected by Ebola [16].

Malawi, despite being one of the poorer countries in SSA, has seen great success in their efforts to increase the percentage of women giving birth in health facilities [17,18]. Despite increased facility-based births, the maternal mortality ratio (MMR) in Malawi remains high at 349/100,000 deaths compared to the desired MMR of 70/100,000 as per the Sustainable Development Goals by 2030. Additionally, the country’s neonatal mortality rate of 27/1000 live births are far from the desired 12/1000 by the year 2030 [19].

In the early days of the COVID-19 pandemic, Malawi implemented a 21-day lockdown starting April 18, 2020. Part of the lockdown included restricted public transportation to health workers and a broadly defined “emergencies only” which included birth-related needs [20]. Due to the shortage of personal protective equipment (PPE) and safety concerns, healthcare workers at the country’s largest medical center went on a two-week strike in April 2020 [20]. During these strikes, patients were turned away, and women were forced to give birth at home [20]. To date, it is unclear how these health system disruptions may have affected maternal and neonatal health in Malawi, as there is a dearth of research around this topic.

Therefore, this study aims to determine if there was a decrease in the number of births in health facilities and if there was a difference in the number of complications reported during facility-based births pre- and post-COVID-19 lockdown.

## Materials and methods

This study was embedded in a larger project based at the University of California San Francisco (UCSF) with the Global Action in Nursing (GAIN) partnership. GAIN currently provides intensive training and longitudinal mentorship to seven health facilities out of 28 in Blantyre district, Malawi.

This study focused on the impact of the COVID-19 pandemic on select maternal and neonatal outcomes in GAIN partnering facilities in Blantyre District Malawi. It included data over a period of 24 months, from 15 months before COVID-19 emerged, defined as the pre-Covid period (January 2019 –March 2020) with nine months after COVID-19 was declared a global pandemic, defined as the initial-covid period (April 2020 –December 2020).

### Study population

**Fig 1:**
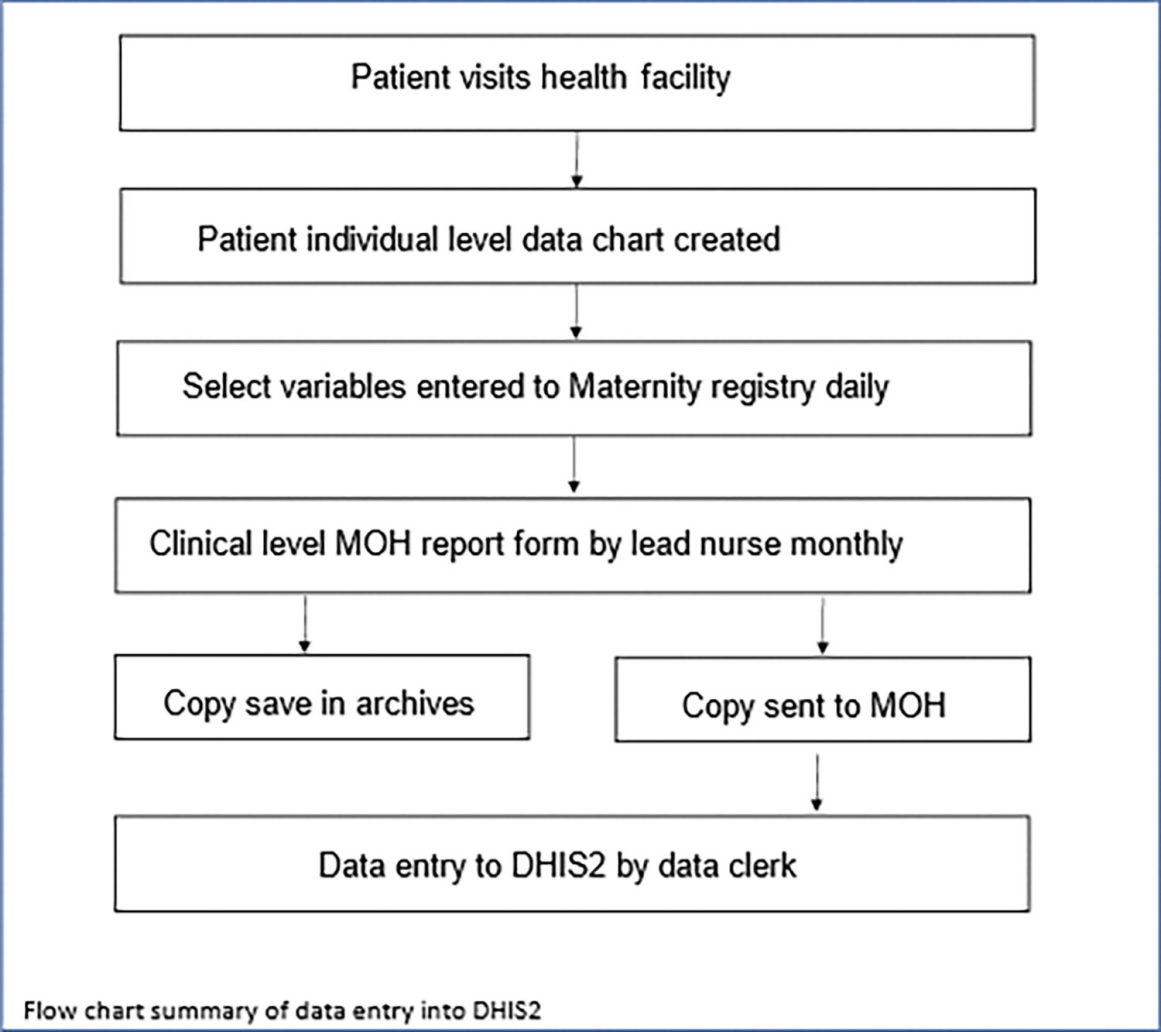
Process summary of patient data collection and entry into DHIS2.

The study population consisted of mothers and their babies born at Bangwe, Limbe, Ndirande, South Lunzu and Zingwangwa health facilities in Blantyre district during the timeframe listed above. The sample included de-identified data from all mothers and babies that attended these health facilities over the study period through standardized aggregate patient monthly summary reports with select variables pre-determined by the Ministry of Health (MoH) seen in the S1 File. The data were initially collected from a patient clinical record after the clinic visit. Information from the patient visit was then recorded daily into a maternity registry that was compiled into a monthly report, which is then shared with the MoH via the district data entry clerks to input into DHIS2 (Fig 1). The District Health Information Software (DHIS-2,) an open-source software provided to and used by many MoHs in SSA and elsewhere, is used to track relevant health indicators. Access to its data is available via the approval of the Malawian MoH. Over the two-year study period we condensed the summary reports into 120 observation periods (five health facilities over 24 months) and the data contained 21,178 births.

Our inclusion criteria consisted of all the data collected from the health facilities over the study time period. Data was first checked for completeness, with missing variables or those thought to be erroneous (defined as two standard deviations from the facility’s mean for a given variable) double checked against facility hard-copies of the reports by GAIN staff. Cells were excluded if the entry could not be located to address or verify missing data and outliers.

### Study variables

Our variables were limited to those that are part of routine data collection from the facilities and recorded via monthly maternity report to the data DHIS2, with all reported maternal and neonatal variables reported in the results. These are grouped by the MoH into broad categories relating to referral, delivery route, obstetric complication, emergency obstetric care, neonatal complication, and perinatal death. Maternal deaths are reported via a separate route and so were not available during the study. The main variable of interest was the presence of COVID-19 and, for this designation, we chose our time frame as a 15-month period before COVID-19 was declared a pandemic by WHO (pre-COVID) and nine months after the declaration of the pandemic (COVID).

This study evaluated all the variables that were captured in the monthly report (Table 1), with special attention paid to the variables that were already known to be related to COVID-19 in the literature, such as referral to advanced care, c-section, and prematurity [21]. The variables were captured as absolute numbers from each clinic, which were limited by the number of actual births at a given month. Therefore, we changed the absolute numbers of each variable into a percentage of births per month, rather than having them as discrete numbers for the sake of comparison.

**Table 1:** Broad categories and sub-categories of maternal and neonatal outcome variables of interest.

Delivery route	Obstetric complications	Emergency Obstetric Care	Neonatal complications	Perinatal death	Referral to care
Spontaneous vertex (Vaginal)	Ante-Partum Hemorrhage (APH)	Antibiotics	Total neonatal complications	Total deaths	Total patient referrals
Vacuum extraction	Fetal Distress	Anti-Convulsants	Asphyxia		
Breech	Any other direct obstetric complication	Blood Transfusion	Other newborn complications		
Caesarean section	partum Hemorrhage (PPH)	Oxytocin	Prematurity		
Spontaneous vertex (Vaginal)	Pre-eclampsia or eclampsia	Manual removal of placenta	Low birthweight, less than 2500g		
Vacuum extraction	Premature Labor		Sepsis		
Breech	Obstructed or Prolonged Labor				
	Retained Placenta				
	Ruptured Uterus				
	Postpartum Sepsis				

### Data analysis

All quantitative analysis was conducted using R statistical software [22]. Data is reported first using descriptive methods for both pre- and COVID periods. Next, bivariate analyses were conducted to assess distributions of maternal and neonatal health variables based on the COVID status. Due to the non-normal distribution of the percentages of interest, the non-parametric Wilcox Rank-Sum was used with a significance cutoff of *p<0*.*05*. The variable for provision of oxytocin for emergency obstetric care was excluded from our study due to known discrepancies in interpretation of reporting requirements based on its usage (“emergency obstetric care” or to manage partum hemorrhage) [23]. Given the importance of placing findings in a local context, two GAIN expert midwife mentors (RM &LS) worked with the study team to ground the design, analysis, and interpretation of the study in the lived experience of the population of interest.

### Ethical considerations

The study was embedded in a larger ongoing study which already had IRB approval from the Malawi National Health Science Research Committee (NHSRC) and the UCSF Committee for Human Research (IRB #18–26842, NHSRC #294890). As the study comprised secondary amalgamated data captured in DHIS2, no consent was required.

## Results

This study included data on 21,143 births from January 2019 to December 2020 through 120 monthly facility reports at five health facilities in Blantyre, Malawi. This included a total of 13,065 births (61.8%) from 75 monthly reports in the pre-COVID period and 8,078 births (38.2%) from 45 monthly reports in the COVID period. The health facilities accommodated 201 births per month on average during pre-COVID and 180 births per month in the COVID period.

Table 2 summarizes where the delivery happened, at the facilities, in transit, assisted by TBA or other facilities outside of the participant facilities during the pre- and COVID-19 period. This study did not find any significant change in the mode of delivery. However, there was a significant decrease in the delivery of mothers assisted by (TBAs) in the Covid period from 1.45% to 0.72% (p = 0.04).

**Table 2:** Summary of place of maternal delivery variables both pre and post COVID-19 period showing the absolute number of occurrences (n) as well as the median percent and IQR for their frequency at the participating facilities.

	Pre-COVID			COVID			P-value
	n	Median%	IQR	n	Median%	IQR	
Total number of deliveries*	13,065	-	-	8,078	-	-	-
Delivery at all facilities	12,490	96.38	(94.95–98.61)	7773	96.89	(95.12–98.40)	0.46
Delivery in transit	285	2.10	(0.86–3.31)	139	1.57	(0.76–2.46)	0.26
Delivery by birth attendants	230	1.45	(0.70–2.44)	96	0.72	(0.00–1.97)	0.04
Delivery at other facilities	10	<0.01	(0.00–0.00)	8	<0.01	(0.00–0.00)	0.51

During the entire study period, spontaneous vaginal deliveries (SVD) were the most common mode of delivery, representing 99.0% of all births at participating facilities during the study period, followed by breech births (0.69%), cesarean sections, and vacuum deliveries (<0.01%). Prolonged labor (5.29%) was the highest reported obstetric complication, followed by pre-eclampsia (2.64%), premature labor (2.09%), and postpartum hemorrhage at 1.52%. All other obstetric complications which were not captured in detail accounted for 10.7% of all reported cases. Conversely, complications such as retained placenta, ruptured uterus, and postpartum sepsis were the least reported complications. Among neonatal complications, low birth weight less than 2500g (3.35%) and asphyxia (3.05%) were the most reported neonatal complications.

Table 3 shows a summary of the absolute number of occurrences for the variables of interest, both pre- and during COVID-19, as well as the median and IQR for the percent of occurrence. Significantly fewer women delivered via a traditional birth attendant during the COVID period than prior, dropping from 1.45% to 0.72% (p = 0.04). There was a significant change in vacuum extraction, which decreased from <0.01% in the pre-Covid period to roughly 0% in the COVID period (p = 0.01). The proportion of births reporting fetal distress almost tripled from 0.46% to1.36% (p = 0.001) during the COVID period. There was no significant change seen in SVD, breech, and cesarean section delivery routes. Even though obstructed labor and pre-eclampsia were the highest reported complications, they remained the same or with no significant difference in pre- and COVID-19 period.

**Table 3:** Summary of the variables of interest both pre and post COVID showing the absolute number of occurrences (n) as well as the median percent and IQR for their frequency at the participating health.

	Total			Pre-COVID			COVID			P-value
	n	Median %	(IQR)	N	Median%	IQR	n	Median%	IQR	
**Total number of deliveries** *	21,143	-	-	13,065	-	-	8,078	-	-	-
**Referral to advanced care**	6,307	30.05	(21.83–37.16)	3,885	31.38	(22.9–37.60)	2,442	28.57	(21.43–35.98)	0.54
**Delivery route**										
Spontaneous vertex (Vaginal)	20,687	99.01	(97.2–99.54)	12,790	99.04	(97.27–99.53)	7,897	98.94	(96.91–99.58)	0.77
Breech	181	0.69	(0.00–1.34)	103	0.67	(0.00–1.10)	78	0.76	(0.32–1.41)	0.31
Caesarean section**	247	2.76	(0.00–5.20)	144	<0.01	(0.00–5.40)	103	3.08	(0.00–3.61)	0.99
Vacuum extraction	28	<0.01	(0.00–0.00)	28	<0.01	(0.00–0.00)	0	-	-	0.01
**Obstetric complication**										
Obstructed or Prolonged Labor	1,120	5.29	(3.47–6.94)	666	5.37	(3.35–6.71)	454	5.29	(4.26–7.43)	0.35
Pre-eclampsia or eclampsia	644	2.64	(0.91–4.21)	366	2.56	(0.85–4.01)	278	2.64	(1.20–4.43)	0.65
Premature Labor	669	2.09	(0.00–4.79)	344	1.28	(0.00–4.50)	325	3.08	(1.93–4.82)	0.01
partum Hemorrhage (PPH)	337	1.52	(0.56–2.46)	194	1.26	(0.53–2.23)	143	1.79	(0.59–2.84)	0.25
Ante-Partum Hemorrhage (APH)	268	0.70	(0.00–2.04)	106	0.68	(0.00–1.01)	72	0.62	0.00–1.35)	0.96
Fetal Distress	178	0.65	(0.00–1.12)	150	0.46	(0.00–1.81)	118	1.36	(0.58–2.18)	0.01
Retained Placenta	90	<0.01	(0.00–0.67)	41	<0.01	(0.00–0.42)	49	0.56	(0.00–1.03)	<0.01
Postpartum Sepsis	34	<0.01	(0.00–0.00)	27	<0.01	(0.00–0.38)	7	<0.01	(0.00–0.00)	0.13
Ruptured Uterus	10	<0.01	(0.00–0.00)	10	<0.01	(0.00–0.00)	0	<0.01	(0.00–0.00)	0.17
Any other direct obstetric complication	2,381	10.71	(7.89–16.42)	1,477	12.93	(7.32–16.64)	904	9.89	(7.94–15.70)	0.37
**Emergency obstetric care**										
Antibiotics	242	0.58	(0.00–1.72)	113	0.45	(0.00–1.42)	129	1.06	(0.00–2.79)	0.01
Anti-Convulsants	179	0.53	(0.00–1.40)	67	<0.01	(0.00–0.90)	112	1.25	(0.51–2.17)	<0.01
Blood Transfusion	12	<0.01	(0.00–0.00)	2	<0.01	(0.00–0.00)	7	<0.01	(0.00–0.00)	0.88
Manual removal of placenta	9	<0.01	(0.00–0.00)	8	<0.01	(0.00–0.00)	4	<0.01	(0.00–0.00)	0.84
**Neonatal complications**										
Total neonatal complications	2,168	10.39	(7.51–12.93)	1,283	9.66	(6.67–12.53)	885	11.25	(8.27–13.97)	0.12
Low birthweight, less than 2500g	790	3.35	(2.08–4.90)	459	3.00	(2.02–4.86)	331	3.7	(2.35–5.26)	0.29
Asphyxia	681	3.05	(1.88–4.17)	368	2.80	(1.48–3.76)	313	3.45	(2.20–5.04)	0.01
Prematurity	421	1.69	(0.85–2.88)	270	1.86	(1.06–2.81)	151	1.55	(0.68–2.88)	0.42
Other newborn complications	244	0.98	(0.49–1.55)	162	1.12	(0.47–1.75)	82	1.01	(0.50–1.36)	0.19
Sepsis	32	<0.01	(0.00–0.00)	24	<0.01	(0.00–0.08)	8	<0.01	(0.00–0.00)	0.08
Perinatal death	125	0.53	(0.00–0.97)	77	0.51	(0.00–0.97)	48	0.55	(0.00–0.81)	0.76

* Total number of deliveries at all participating was used to as a denominator to compute all maternal and neonatal variables.

** C-section was analyzed based on only two health facilities (Ndirande and Bangwe) as those are the only health facilities that perform C-sections.

Additionally, the proportion of births reporting anti-convulsant use increased from 0.01% to 1.2% (p<0.01), and antibiotic use increased from 0.45% to 1.6% (p = 0.01) during the COVID-19 period. Asphyxia was the only significant neonatal complication variable reported, which increased from 2.80% to 3.45% (p = 0.01).

## Discussion

The primary purpose of this study was to assess whether fewer women gave birth in facilities during the covid period as well as whether the emergence of the disease was linked with changes in reported maternal and neonatal outcomes. This discussion is framed by a combination of the study findings, existing literature, and the opinion of experts working in the study health facilities.

Despite evidence from elsewhere suggesting that women would avoid or be unable to access facilities, surprisingly there was a significant decline reported in TBA-assisted delivery during the COVID-19 period. This may be associated with the enforced social distancing and restricted transportation to only front-line workers. It also could be that the lockdowns lifted other barriers to care such as access to ambulances due to fewer road-traffic and other external injuries.

This study found a significant decrease of vacuum delivery in the COVID-19 period. While there is no established literature currently showing an association between COVID-19 and changes in vacuum delivery, the expert mentors interviewed for this study attribute this to both midwives avoiding use of vacuum delivery systems as well as a shortage of experienced midwives who were comfortable using vacuum as a delivery method. Challenges such as this highlight the benefits of continuing professional development courses on vacuum extraction for all midwives as a useful adjunct to current in-service refresher trainings.

We found a significant increase in fetal distress during the COVID period. While this is has not been shown to be causally linked to COVID in the literature, the study expert mentors, the increase found in this study may have been due to staff shortage during the post COVID-19 period and associated issues in maternal care [24]. Additionally, they noted that mothers with complications expressed hesitancy at being referred to higher level of care due to limited transportation caused by district lockdown restrictions and fears of contracting COVID at the hospitals. This saw mothers who normally would have been transferred to a referral hospital and delivering in the health facility instead. It is heartening to see that despite these increases in fetal distress, there was no significant increase in neonatal death.

Premature birth was also significantly higher in the COVID-19 period in our study sample. This is interesting as emerging literature for developed countries showed declines in preterm birth overall following COVID lockdowns, though increases among those who were infected [25–27]. The expert midwives attributed the increased preterm labor during the COVID-19 period to potential stress mothers could have faced in the start of the pandemic as well as the potential that the mothers had been infected with COVID. Due to the limited testing capacity in developing regions, especially during the start of the outbreak the association cannot be directly made, and further research is warranted.

The increase in anticonvulsant usage during COVID-19 period was higher compared to the pre-COVID-19 period and may be associated with untreated malaria and uncontrolled preeclampsia, both known to cause seizures and potentially exacerbated due to COVID restrictions [28,29]. These indirect barriers include disruption in antenatal care, mothers missing antenatal care from fear of going to health facilities, the closure of healthcare facilities, or lack of transportation services. This tracks with a study done in Uganda showing a decrease in detection rate of malaria during the COVID-19 pandemic [30]. Given that Malawi is an endemic malarial region, this is an important factor to consider especially as pregnant women with malarial infections may not have been treated or received intermittent preventive therapy (IPTp), likely due to indirect effects of COVID-19. Hence, mothers who were not coming to antenatal care and those who failed to get their medication were potentially at a higher risk of experiencing a seizure. It is also possible women who missed antenatal care visits also missed blood pressure checkups to screen for pre-eclampsia before they became eclamptic. However, antenatal visits were not captured in the maternal outcome DHIS dataset, so the study was not able to detect a decline in antenatal care visits. Further research could explore relationships between these variables.

The significant increase in the usage of antibiotics during the COVID-19 period is limited in interpretation due to the lack of ability to track infection in patients. It may be that patients presenting with COVID symptoms were given antibiotics, which would fit patterns of antibiotic prescription practices in the region [31,32].

Asphyxia was the only reported neonatal complication that was significantly higher in the COVID-19 period. A systematic review conducted from January through March 2020 assessed the effect of COVID-19 on pregnancy in China and found 1.2% of births reported neonatal asphyxia. However, the study did not find a significant difference in pre and post COVID-19 period [24]. The expert mentors interviewed for this study hypothesized that the neonates born to unattended mothers–due to worker shortages at facilities—with prolonged labor may have experienced asphyxia more frequently in the COVID-19 period.

Importantly, understanding the impact of COVID-19 on maternal death would be an indicator of interest. However, in this study, we could not explore maternal death as a variable because the study health facilities refer all complicated cases to the district referral hospital. Health facilities do not document obstetric outcomes once the patient is referred, and maternal deaths are captured in a secondary database not accessible to the current study. Currently, this study team is working on a referral project that creates a feedback loop between referral hospitals and the referring health facilities.

## Limitations

The reliance on DHIS2 limited our ability to assess the impact of COVID-19 on each individual patient. Additionally, this study relies on data that is three times removed from the actual site itself, so the likelihood of data error was very high. However, we worked local expert mentors to reduce the potential errors by going to the original data sources and rectifying them manually when discrepancies were noted.

Given that we used summary variables found in DHIS2, some variables, such as antenatal care visits and maternal death, were not captured. Therefore, using those summary variables limited the study’s ability to have a more nuanced understanding of the impact of COVID-19 on obstetric outcomes. We sought to address this by including the experiences of midwife mentors who practiced during the period to provide greater context.

Additionally, we acknowledge that the bivariate relationships of the results are likely confounded by many other factors outside of COVID-19. Normally we would model a multivariable model to control for the possible confounders, however, our variables lacked homoscedasticity, one of the requirements for a multivariable linear regression. The goal of our study is to provide an initial survey into the topic; therefore, based on our initial findings we suggest further research to explore the impacts of COVID-19 on maternal and neonatal health outcomes in depth using patient-level data.

## Conclusion

This is one of the first studies to explore the impact of COVID-19 on maternal health-seeking behavior and outcomes in an LMIC. Our findings suggest that significant outcomes were mainly due to the indirect effects of COVID-19 rather than the virus itself. Based on our findings and the contextual qualitative interviews with Malawian expert midwives, we concluded that mothers may have been affected most by understaffing and shortage of skilled personnel in the study health facilities. Therefore, the financial support of educational advancement for current and new nurse midwives and preceptorship once they start work in the health facilities may contribute to better outcomes in the future, along with adequate staffing and a streamlined referral process. Clear areas for future research were identified that can further shed light on areas for quality assessment and improvement projects.

## Supporting information

S1 File Maternity monthly report to DHIS2. (DOCX)
