# Supplementary material for: Exploring the impact of COVID-19 on reported maternal and neonatal complications and access to maternal health care in five government health facilities in Blantyre, Malawi
Source: PLoS One. 2023 May 23;18(5):e0285847. doi: 10.1371/journal.pone.0285847 (PMC10204969; doi:10.1371/journal.pone.0285847)
Supplement: S1 File — (DOCX) [file pone.0285847.s001.docx]

**S1 .** Maternity Monthly Report to DHIS2
